# Drying Temperature Precision Control System Based on Improved Neural Network PID Controller and Variable-Temperature Drying Experiment of Cantaloupe Slices

**DOI:** 10.3390/plants12122257

**Published:** 2023-06-09

**Authors:** Taoqing Yang, Xia Zheng, Hongwei Xiao, Chunhui Shan, Xuedong Yao, Yican Li, Jikai Zhang

**Affiliations:** 1 College of Mechanical and Electrical Engineering, Shihezi University, Shihezi 832003, China; 20212009002@stu.shzu.edu.cn (T.Y.);; 2 Key Laboratory of Northwest Agricultural Equipment, Ministry of Agriculture and Rural Affairs, Shihezi 832003, China; 3 Key Laboratory of Modern Agricultural Machinery Corps, Shihezi 832003, China; 4 College of Engineering, China Agricultural University, 17 Qinghua Donglu, Beijing 100083, China; 5 College of Food, Shihezi University, Shihezi 832003, China

**Keywords:** drying temperature, control system, neural network, PID controller, cantaloupe slices, variable-temperature

## Abstract

A drying temperature precision control system was studied to provide technical support for developing and further proving the superiority of the variable-temperature drying process. In this study, an improved neural network (INN) proportional–integral–derivative (PID) controller (INN-PID) was designed. The dynamic performance of the PID, neural network PID (NN-PID) and INN-PID controllers was simulated with unit step signals as an input in MATLAB software. A drying temperature precision control system was set up in an air impingement dryer, and the drying temperature control experiment was carried out to verify the performance of the three controllers. Linear variable-temperature (LVT) and constant-temperature drying experiments of cantaloupe slices were carried out based on the system. Moreover, the experimental results were evaluated comprehensively with the brightness (*L* value), colour difference (Δ*E*), vitamin C content, chewiness, drying time and energy consumption (EC) as evaluation indexes. The simulation results show that the INN-PID controller outperforms the other two controllers in terms of control accuracy and regulation time. In the drying temperature control experiment at 50 °C–55 °C, the peak time of the INN-PID controller is 237.37 s, the regulation time is 134.91 s and the maximum overshoot is 4.74%. The INN-PID controller can quickly and effectively regulate the temperature of the inner chamber of the air impingement dryer. Compared with constant-temperature drying, LVT is a more effective drying mode as it ensures the quality of the material and reduces the drying time and EC. The drying temperature precision control system based on the INN-PID controller meets the temperature control requirements of the variable-temperature drying process. This system provides practical and effective technical support for the variable-temperature drying process and lays the foundation for further research. The LVT drying experiments of cantaloupe slices also show that variable-temperature drying is a better process than constant-temperature drying and is worthy of further study to be applied in production.

## 1. Introduction

As a necessary processing step for the safe storage of agricultural products, drying plays a crucial role in enhancing global food safety [1,2]. In addition, drying can extend shelf life and increase the availability of fresh agricultural products [3]. Microbial contamination and post-harvest losses owing to inadequate drying and backward drying technology are extremely serious, making research into new drying processes and technologies particularly urgent [4].

Variable-temperature drying is a method of drying products at different drying temperatures in various drying stages. This method can increase the drying speed, reduce energy consumption and improve product quality [5]. The variable-temperature drying process has been applied to yam [6], garlic [7], lotus root [5], peanut [8], carrot [9] and other crops. The existing variable-temperature drying process is mainly based on the variable-temperature gradient [10]. Variable-temperature drying processes, such as linear variable temperature (LVT) [9] and variable temperature with a controlled drying rate [11], have more potential advantages. These studies on the more complex variable-temperature drying process will provide a theoretical basis for the drying industry in improving the quality of dried products and reducing the production cost. However, these variable-temperature drying processes have not been widely promoted. The reason is that the target value of the drying temperature needs to be constantly adjusted according to the drying stage and the state of the material, which has higher requirements for the temperature control system. In addition, the non-linearity, hysteresis and time-varying characteristics of the drying temperature control make the temperature control of the relatively complicated variable-temperature drying process a major problem, which also seriously restricts the development of variable-temperature drying processes. Thus, providing technical support for further research of the variable-temperature drying process and studying a drying temperature precision control system are necessary.

Most current drying temperature control systems are constant-temperature control systems [12,13,14]. However, drying temperature control systems for variable-temperature drying processes have rarely been reported. Given the specificity of variable-temperature control, the current drying temperature control systems are no longer suitable for variable-temperature drying processes. A proportional–integral–derivative (PID) controller is a mature and effective algorithm for the linear combination of system errors and is fed back to the controlled object. However, the proportional, integral and derivative parameters of the PID controller need to be pre-set according to human experience, and the control effect is subject to the subjective decision. The PID controller cannot achieve real-time adjustment of the controller parameters and lacks control flexibility. The control effect is hardly satisfactory for control systems with complex non-linearity [15]. In this case, the intelligent PID algorithm, which can adjust the parameters of the PID controller in real time, has emerged. Fuzzy PID [16], neural network PID [17], genetic algorithm PID [18] and particle swarm optimisation PID [19] have been used in various control industries. Neural networks are widely used for the parameter tuning of PID controllers owing to their unique learning ability and non-linear processing capability. However, the convergence speed of neural networks is slow, and the training process of neural networks can easily fall into local optimal rather than global optimal [20]. If the training process of the neural network is improved, then the ability of the neural network to regulate PID parameters can be significantly improved.

Training a neural network means continuously optimising the weights [21]. The initial weights are randomly generated, and the training of neural networks is sensitive to the initial weights, which can easily fall into a local optimum if the initial weights are not chosen appropriately. The genetic algorithm has good global search capability. The genetic algorithm optimises the initial weights of the neural network, and the neural network is trained with the optimised weights, so that the training process can jump out of the local optimum [22]. In addition, the step size is fixed during the training of the neural network. In the early stages of neural network training, a too-small step size will reduce the convergence speed of the neural network. In the later stages of neural network training, a too-large step size will cause oscillations. Therefore, the training step size of the neural network needs to be adjusted in real time. Furthermore, the training process of neural networks does not incorporate the experience of previous iterations, which reduces the convergence speed and the accuracy of the training results. In the end, the input parameters of the neural network in the existing research do not fully reflect the real state of the system, which also greatly affects the training results of the neural network. Suppose the training process of the neural network is improved in light of the problems above and a drying temperature precision control system is designed based on the improved neural network (INN) PID controller (INN-PID). In this case, the temperature in the variable-temperature drying process can be adjusted quickly and effectively.

Air impingement technology is a drying technology, where pressurised hot air is ejected through a nozzle, and the impact and heating of the hot air on the material takes away the moisture. High drying speeds and high heat transfer coefficients characterise the technology. A drying temperature precision control system is built on this equipment, which can be combined with the characteristics of the equipment to further improve the quality of the dried product. Cantaloupe has a high moisture content and short shelf-life and is prone to breakage during the picking and distribution process, leading to mould contamination. Drying is a common method to extend the shelf life of cantaloupes [23]. In this study, the LVT drying of cantaloupe slices was carried out and compared with constant-temperature drying in terms of energy efficiency and quality of the dried product. The advantages of the LVT drying process over constant-temperature drying were obtained, further demonstrating the effectiveness of the former process.

In summary, this study aims (1) to build a temperature control system in an air impingement dryer and establish a mathematical model for temperature control of the system; (2) to complete the theoretical derivation of the PID, neural network PID (NN-PID) and INN-PID algorithms; (3) to simulate the dynamic performance of the controllers of the above three algorithms using MATLAB software and analyse the simulation results; (4) to conduct experiments on the drying temperature control of the three controllers and analyse the temperature control effect of the three controllers; and (5) to conduct LVT drying experiments on cantaloupe slices; the brightness (*L* value), colour difference (Δ*E*), chewiness, vitamin C content, drying time and energy consumption (EC) were used as evaluation indexes, and a comparison analysis with a constant-temperature drying experiment was made. This study will provide effective technical support to further development of the variable-temperature drying process and further verify the superiority of the variable-temperature drying process.

## 2. Materials and Methods

### 2.1. Structural Components of the Drying Temperature Precision Control System

**Figure 1:**
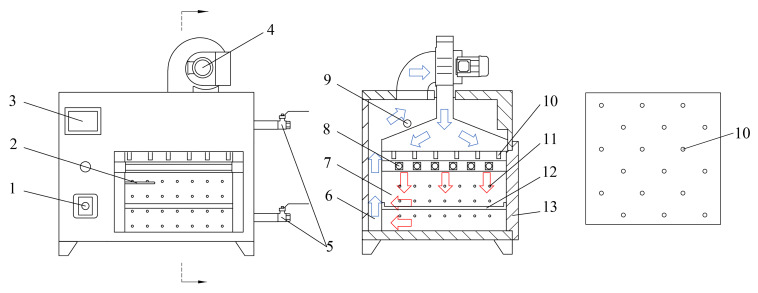
The principal diagram of the air-impingement dryer. (1) Air velocity adjustment knob; (2) temperature transducer; (3) temperature control touch panel; (4) centrifugal fan; (5) wet discharge valve; (6) drying outer chamber; (7) drying inner chamber; (8) infrared heating tube; (9) weephole; (10) air nozzle; (11) air outlet; (12) drying tray; (13) door.

Figure 1 shows the principal diagram of the air impingement dryer. The equipment consists of a fan, infrared heating tube, temperature sensor, wet discharge valve and other devices. The dryer is divided into two parts: the inner and outer chambers. The fan circulates the air between the inner and outer chambers. The hot air heated by the infrared heating tube passes through the nozzle and becomes a high-speed hot air stream, affecting the tray’s material. The material is dried under the combined effect of the high temperature and airflow. The hot air from the inner chamber enters the outer chamber through the air outlet. The moist air in the outer chamber is discharged from the wet discharge valve. The remaining air is sucked in by the fan and blown into the inner chamber. The air is circulated internally between the inner and outer chambers, avoiding heat loss and reducing EC.

A temperature sensor is used to detect the temperature of the inner chamber. The temperature of the inner chamber is regulated by controlling the power of the six infrared heating tubes. An increase in air velocity can significantly increase the drying rate of the material [21]. As the fan power is a small proportion of the total power of the equipment, an increase in air velocity does not greatly increase the total power of the equipment. Regarding drying efficiency and EC, the fan is operated at a maximum air velocity of 4 m/s. With a weep hole internal diameter of 15 mm, the air exchange between the outer chamber and the outside world has a low effect on the internal air circulation within the appliance. Thus, the wet discharge valve is usually opened to the maximum. As the outer chamber is not heated by the infrared heating tubes, the temperature of the outer chamber is significantly lower than that of the inner chamber. In the air circulation between the inner and outer chambers, controlling the temperature in the inner chamber is more difficult owing to the temperature difference.

The STM32 microcontroller was chosen as the control element. The INN-PID control algorithm is written into the microcontroller. The microcontroller calculates the target value of the drying temperature at the current time based on the variable-temperature mode. The target value of the drying temperature is used as the desired value, and the actual temperature collected by the temperature sensor is the feedback value. Moreover, the microcontroller calculates the control quantity through the algorithm to control the power of the infrared heating tube and finally realise the accurate control of the drying temperature.

The material is placed on a tray for variable-temperature drying, and the chamber door is closed. The fan works at a maximum air velocity of 4 m/s, and the opening of the wet discharge valve is at its maximum. The system calculates the set value of the current temperature. When the temperature sensor detects a deviation between the internal chamber temperature and the set value, the system automatically adjusts the power of the infrared heating tube to maintain a stable state.

Establishing a mathematical model for temperature control in the air impingement dryer is a prerequisite for studying this system. The first-order inertia plus delay link transfer function is chosen to describe the mathematical model of this temperature control system based on the complexity of the system and the characteristics of the temperature control.

(1) $$G(s)=\frac{K}{Ts+1}e^{-\tau_{1}s}=\frac{1.4}{76.4s+1}e^{-25s}$$

The sampled data from this system are fitted using a first-order approximation to more accurately establish a mathematical model of a drying temperature precision control system. The temperature of the inner chamber is sampled at an interval of 1 s using the target value of the temperature control as an input to the open-loop system. The gain coefficient *K* of the system is 1.4, the delay time is 25 s and the time constant *T* is 76.4. The mathematical model of the system is shown in Equation (1).

### 2.2. Design of an INN-PID Controller

#### 2.2.1. Design of a Conventional PID Controller

**Figure 2:**
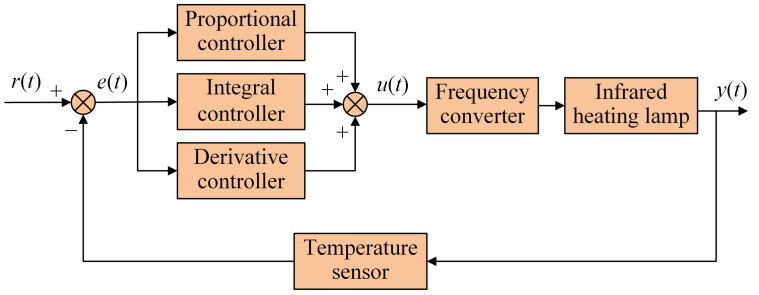
Structure of a conventional PID controller.

Conventional PID controllers operate through the interplay among three units: proportional, integral and derivative. As one of the earliest control strategies, the PID controller is easy to adjust, simple in structure and widely used in industrial process control [24]. Figure 2 shows the structure of a conventional PID controller.

Where *r*(*t*) is the desired value, *y*(*t*) is the actual output value and *e*(*t*) is the deviation between the desired and actual output values. The proportional, integral and derivative units output the control quantity *u*(*t*) according to *e*(*t*). *u*(*t*) acts on the controlled object so that the actual output value *y*(*t*) is constantly close to *r*(*t*), thereby realising the regulation of the controlled object. The mathematical expression for the control quantity *u*(*t*) is as follows:

(2) $$u(t)=K_{p}\left[e(t)+\frac{1}{T_{i}}\int_{0}^{t}e(\tau )d\tau +T_{d}\frac{de(t)}{dt}\right]$$

where *K_p_* is the scale factor, *T_i_* is the integration time constant and *T_d_* is the derivative time constant.

Considering that sampling and control are discrete-time signals, the continuous time *t* is divided into *k* sampling periods *T*. The PID equation is as follows:

(3) $$\begin{array}{r} u(k)=K_{p}e_{k}+K_{p}\frac{T}{T_{i}}\sum_{j=0}^{k}e_{j}+K_{p}\frac{T_{d}}{T}\left(e_{k}-e_{k-1}\right) \end{array}=K_{p}e_{k}+K_{i}\sum_{j=0}^{k}e_{j}+K_{d}\left(e_{k}-e_{k-1}\right)$$

where *K_i_* is the integration factor and *K_d_* is the derivative factor; $K_{i}=K_{P}\frac{T}{T_{i}}$,$K_{d}=K_{P}\frac{T_{d}}{T}$.

In Equation (3), *u*(*t*) is related to every deviation *e* from time 0 to *k*. When a computer anomaly causes a large change in *y*(*k*) or *r*(*k*), eliminating the large deviation *e*(*k*) in a short time is difficult, seriously affecting the stability of the system. In this case, the PID equation can be expressed in incremental form.

(4) $$\begin{array}{r} u(k-1)=K_{p}e_{k-1}+K_{i}\sum_{j=0}^{k-1}e_{j}+K_{d}(e_{k-1}-e_{k-2}) \end{array}$$

Subtracting Equation (3) from Equation (4) gives the following:

(5) $$\begin{array}{r} \Delta u(k)=K_{p}(e_{k}-e_{k-1})+K_{i}e_{k}+K_{d}(e_{k}-2e_{k-1}+e_{k-2}) \end{array}$$

The incremental PID is expressed as follows:

(6) $$\begin{array}{r} u(k)=u(k-1)+\Delta u(k) \end{array}$$

When the three parameters of the PID are fixed, the control quantity *u*(*k*) is only related to *e_k_*, *e_k−1_* and *e_k−2_*, and the stability of the system is significantly improved. The above formula derivation shows that the choice of PID parameters greatly affects the performance of the controller. Moreover, in the face of more complex control systems, the PID parameters must be adjusted in real time to meet the requirements of the system.

In this study, the Cohen–Coon method was used to determine the three PID parameters [25], and the rectification equation is shown in Equation (7).

(7) $$\left\{\begin{array}{l} K_{p}=\frac{T}{K\tau }\left(\frac{4}{3}+\frac{\tau }{4T}\right)\\ T_{i}=\tau \left(\frac{32+\frac{6\tau }{T}}{13+\frac{8\tau }{T}}\right)\\ T_{d}=\tau \left(\frac{4}{11+\frac{2\tau }{T}}\right) \end{array}\right.$$

According to Equations (2), (3) and (7), we can obtain *K_p_* = 3.14, *K_i_* = 0.06 and *K_d_* = 0.36.

#### 2.2.2. Design of the Neural Network PID Controller

**Figure 3:**
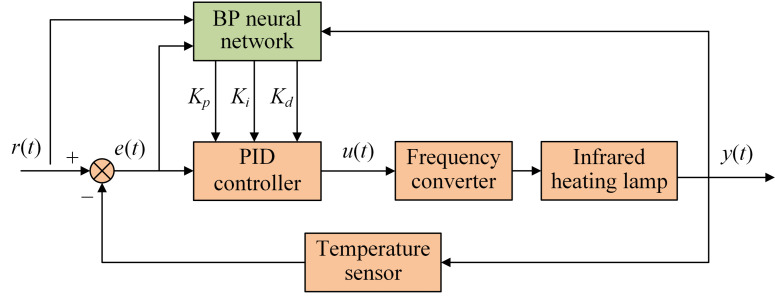
Structure of NN-PID controller.

The neural network with autonomous learning capability can make real-time adjustments to the three parameters of the PID controller according to the state of the system [26]. A backpropagation neural network (BP-NN) is a feedforward network training model based on an error backpropagation algorithm. BP-NN is one of the most widely used neural network models in PID controller parameter adjustment. All the neural networks in this study refer to BP neural networks. The neural network takes the state of the control system (*r*(*t*), *y*(*t*) and *e*(*t*)) as input and outputs the optimal values of the three PID parameters. The conventional PID controller obtains the optimal parameters and, based on the current system error *e*(*t*), outputs the control quantity *u*(*t*) to regulate the controlled object. Figure 3 shows the NN-PID controller.

**Figure 4:**
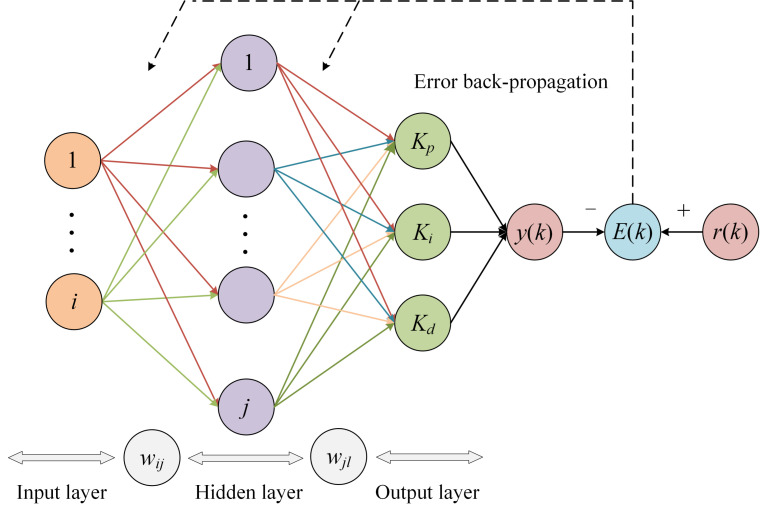
Neural network topology.

The topology of the neural network consists of three layers, namely the input, hidden and output layers. The input layer has three neurons, representing *r*(*t*), *y*(*t*) and *e*(*t*). The hidden layer has five neurons. The output layer has three neurons representing the three parameters of the PID controller. Data transfer between the neural network layers is achieved through weights and activation functions. Figure 4 depicts the topology of the neural network.

The training process of the neural network is particularly important in regulating the PID parameters as it determines whether the PID parameters are optimal and ultimately the output control quantity *u*(*t*). The training process of the neural network is as follows.

The output of the input layer is as follows:

(8) $$O_{i}^{\left(1\right)}=x(i)$$

where *x*(*i*) represents the expected value, the actual value and the deviation in that order.

The input to the hidden layer is $\begin{array}{r} net_{j}^{\left(2\right)} \end{array}$, and the output is $O_{j}^{\left(2\right)}$.

(9) $$\begin{array}{r} net_{j}^{\left(2\right)}(k)=\sum_{i=0}^{M}w_{ij}{}^{\left(1\right)}O_{i}^{\left(1\right)} \end{array}$$

(10) $$O_{j}^{\left(2\right)}(k)=f\left(net_{j}^{\left(2\right)}(k)\right)$$

where $w_{ij}{}^{\left(1\right)}$ is the weight between the *i*-th neuron in the input layer and the *j*-th neuron in the hidden layer. The number of neurons in the hidden layer is set to 5 according to the complexity of the topology. Moreover, the activation function between the input and hidden layers is a positive and negative symmetric Sigmoid function, as shown in Equation (11).

(11) $$\begin{array}{r} f(x)=\frac{e^{x}-e^{-x}}{e^{x}+e^{-x}} \end{array}$$

The input to the output layer is $\begin{array}{r} net_{l}^{\left(3\right)} \end{array}$, and the output is $O_{l}^{\left(3\right)}$.

(12) $$\begin{array}{r} net_{l}^{\left(3\right)}(k)=\sum_{j=0}^{Q}w_{jl}{}^{\left(2\right)}O_{j}^{\left(2\right)} \end{array}$$

(13) $$O_{l}^{\left(3\right)}(k)=g(net_{l}^{\left(3\right)}(k))$$

where $w_{jl}{}^{\left(2\right)}$ is the weight between the *j*-th neuron in the hidden layer and the *l*-th neuron in the output layer. Considering that the output of the output layer *K_p_*, *K_i_* and *K_d_* is non-negative, the activation function of the output layer is chosen as a non-negative hyperbolic tangent function [27], as shown in Equation (14).

(14) $$\begin{array}{r} g(x)=\frac{e^{x}}{e^{x}+e^{-x}} \end{array}$$

The three outputs of the output layer are the three parameters of the PID controller *K_p_*, *K_i_* and *K_d_*.

(15) $$\left\{\begin{array}{l} O_{1}^{\left(3\right)}\left(k\right)=k_{p}\\ O_{2}^{\left(3\right)}\left(k\right)=k_{i}\\ O_{3}^{\left(3\right)}\left(k\right)=k_{d} \end{array}\right.$$

*E*(*k*) is the error function that provides error feedback on the training process of the neural network.

(16) $$\begin{array}{r} E(k)=\frac{1}{2}{\left[r(k)-y(k)\right]}^{2} \end{array}$$

The neural network uses gradient descent to optimise the weights, and the neural network optimises the weights in the direction of the negative gradient of the error function *E* [28]. The formula for optimising the weights between the hidden and output layers is shown in Equation (17).

(17) $$\begin{array}{r} \Delta w_{jl}^{\left(2\right)}(k)=-\eta \frac{\partial E(k)}{\partial w_{jl}^{\left(2\right)}} \end{array}$$

where $\begin{array}{r} \eta \end{array}$ is the learning rate, the iteration step size. Equations (18) and (19) are derived from the chain rule.

(18) $$\frac{\partial E(k)}{\partial w_{jl}^{\left(2\right)}}=\frac{\partial E(k)}{\partial y(k)}\times \frac{\partial y(k)}{\partial \Delta u(k)}\times \frac{\partial \Delta u(k)}{\partial O_{l}^{\left(3\right)}(k)}\times \frac{\partial O_{l}^{\left(3\right)}(k)}{\partial net_{l}^{\left(3\right)}(k)}\times \frac{\partial net_{l}^{\left(3\right)}(k)}{\partial w_{jl}^{\left(2\right)}(k)}$$

(19) $$\begin{array}{r} \Delta w_{jl}^{\left(2\right)}(k)=\eta \delta_{l}^{\left(3\right)}O_{j}^{\left(2\right)}(k) \end{array}$$

The optimised weights between the hidden and output layers are as follows:

(20) $$\begin{array}{cc} w_{jl}^{\left(2\right)}(k+1) & =w_{jl}^{\left(2\right)}(k)+\eta \delta_{l}^{\left(3\right)}O_{j}^{\left(2\right)}(k) \end{array}$$

Similarly, the optimisation weights between the input and hidden layers can be obtained as follows:

(21) $$\begin{array}{r} \Delta w_{ij}^{\left(1\right)}(k)=\eta \delta_{j}^{\left(2\right)}O_{i}^{\left(1\right)}(k) \end{array}$$

(22) $$\begin{array}{cc} w_{ij}^{\left(1\right)}(k+1) & =w_{ij}^{\left(1\right)}(k)+\eta \delta_{j}^{\left(2\right)}O_{i}^{\left(1\right)}(k) \end{array}$$

After the optimised weights are obtained, the next generation is trained until the error function meets the requirements.

#### 2.2.3. Design of the Improved Neural Network PID Controller

This study improves the training process of neural networks to address the problems of slow convergence and poor accuracy of the training process of neural networks. The INN is used to adjust the parameters of the PID controller.

1. Genetic algorithm to optimise initial weights of neural networks

**Figure 5:**
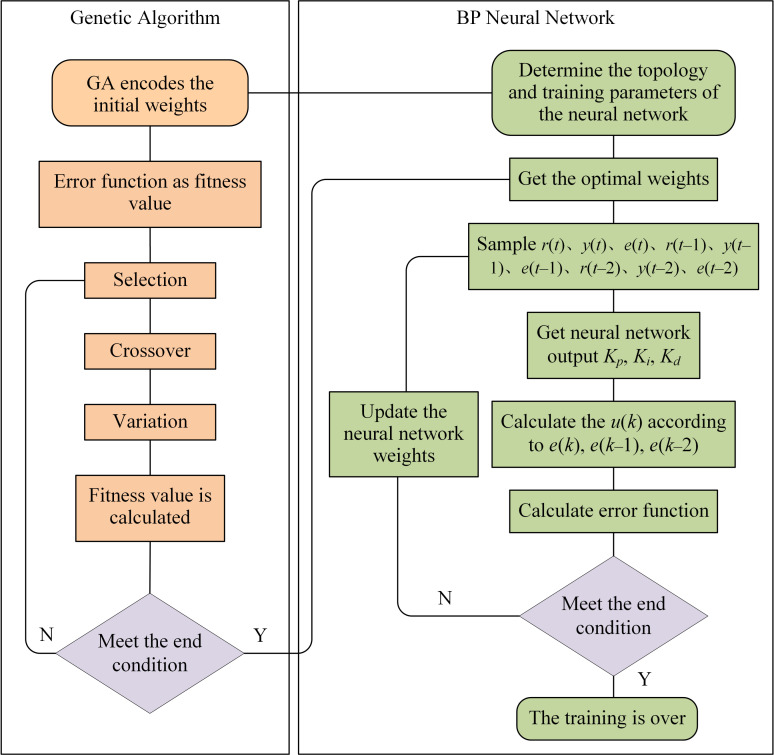
The flow chart of the algorithm.

The choice of initial weights is the key to neural network training and has the most direct influence on the speed of convergence and the accuracy of the training results. The improper selection of initial weights can cause the training process of neural networks to fall into local optima [29]. Genetic algorithms are global optimisation algorithms that simulate the survival of the fittest in biological evolution. The genetic algorithm can make the training process of the neural network out of the local optimum and obtain the global optimum by optimising the initial weights of the neural network. Figure 5 shows the process of optimising the initial weights.

(1) Population initialisation: set population size *P*, crossover probability *P_c_*, mutation probability *P_v_*. Encode initial weights.
(2) The fitness value is calculated for each individual through the fitness function, which is the reciprocal of the error function *E*. The larger the error of an individual, the smaller the fitness. The fitness of the *i*-th individual is expressed as follows:
  (23) $$f_{i}=1/E_{i}$$
(3) Individuals are screened based on a selection operator, and the higher the value of individual fitness, the higher the probability of being selected. The probability is calculated from Equation (24). The screened individuals are randomly paired into pairs, and each pair is crossed over with the crossover probability. The population is selected, crossed and mutated to produce a new population.
  (24) $$p_{i}=\frac{f_{i}}{\sum_{i=1}^{N}f_{i}}$$
(4) The individual fitness values of the new population are calculated. If the average fitness value of the population reaches the iteration termination condition, then the resulting weights are assigned to the neural network. Otherwise, the next iteration is performed.
(5) Neural network training is started based on the weights optimised by the genetic algorithm. Training is stopped when the error function *E*(*k*) reaches a predetermined value.

Figure 5 depicts the flow chart of the algorithm for optimising the initial weights of the neural network with the genetic algorithm.

2. Variable learning rate algorithm

This study proposes a variable learning rate algorithm to accelerate the convergence speed of neural network training and improve the accuracy of neural network models.

The learning rate of neural network η is in the range of [0, 1]. The larger the learning rate, the greater the modification of the weights and the faster the network learning speed. However, a too-large learning rate will make the training process of the neural network oscillate and difficult to stabilise. By contrast, a too-small learning rate will greatly reduce the convergence speed of the neural network and make the training results more likely to fall into the local optimum. The variable learning rate algorithm means that the learning rate is taken to be larger at the beginning of the training of the neural network to speed up the convergence of the network whilst preventing the training results from falling into the local optimum. As the training process progresses and the training results are close to the global optimum, stabilising the training process and avoiding oscillations would be easier by adopting a smaller learning rate. The formula for calculating the variable learning rate is as follows:

(25) $$\eta (t)\;=\;\eta_{max}-t(\eta_{max}-\eta_{min})/t_{max\;}$$

where *η_max_* is the maximum learning rate, *η_min_* is the minimum learning rate, *t_max_* is the maximum number of iterations and *t* is the current number of iterations.

3. Additional momentum method

The neural network uses the gradient descent method to optimise the weights and correct the weights from the negative gradient direction of the error function. The weight correction of the neural network only considers the current error function, ignoring the accumulated experience of the previous weight correction, resulting in slow convergence of the training process. The additional momentum method can be used to solve this problem. The formula of the additional momentum method was represented as follows:

(26) $$\omega (k)=\omega (k-1)+\Delta \omega (k)+a\left[\omega (k-1)-\omega (k-2)\right]$$

where ω(*k*), ω(*k*−1) and ω(*k*−2) are the weights at times *k*, *k*−1 and *k*−2, respectively, and *a* is the inertia factor.

The additional momentum method plays a damping role in training neural networks. The additional momentum method can reduce the oscillation when the training error is abrupt. When the training error remains flat, the additional momentum method will accelerate the convergence rate of the neural network owing to the previous weight correction experience.

4. Neural network input parameter optimisation

**Figure 6:**
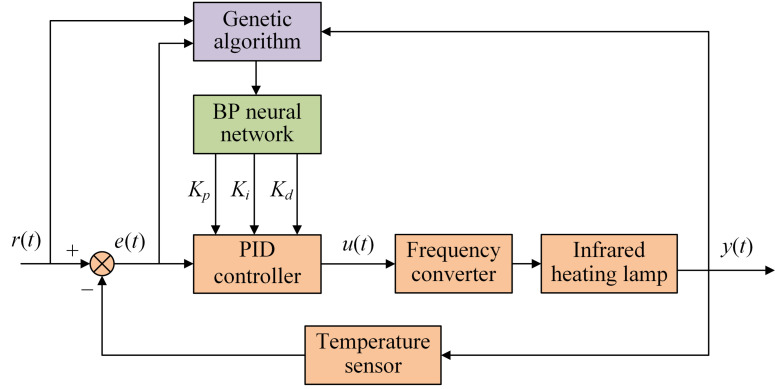
INN-PID controller structure.

From Equations (5) and (6), with the three parameters of the PID fixed, the control quantity 𝑢(𝑘) is related to the deviation of the three moments, namely, *e_k_*, *e_k_*_−1_ and *e_k_*_−2_. Therefore, the state of the system at the three moments needs to be fully considered by making real-time adjustments to the three parameters and outputting the optimal *u*(*k*). In conventional neural network PID algorithms, the neural network has only three inputs, *r*(*t*), *y*(*t*) and *e*(*t*). The conventional algorithm only considers the system state at one moment, which makes making accurate adjustments to PID parameters difficult. This study proposes a new neural network structure. The system state at three moments is the input. The three parameters of the PID controller are the outputs. This neural network structure allows for accurate adjustment of the PID parameters once the system state is fully considered. Figure 6 shows the INN-PID controller structure.

### 2.3. Dynamic Performance Simulation of Three Controllers

Simulations of the controllers for the three algorithms were carried out in MATLAB R2021A software to test the dynamic performance. The step input is a relatively severe operating condition for the system. Moreover, if the dynamic performance of the system under the action of the step function meets the requirements, then the dynamic performance of the system under other forms of input should also be satisfactory [30]. In engineering, peak time, regulation time and maximum overshoot are commonly used as indicators to evaluate the dynamic performance of the system.

Peak time is the time taken for the step response to exceed the target value and reach the first peak. Regulation time is the minimum time required for the step response to reach and remain within the ±5% error band. Maximum overshoot is the percentage by which the maximum peak exceeds the target value.

### 2.4. Experiments with Three Controllers for Drying Temperature

A drying temperature precision control system is constructed using an air impingement dryer. Experiments with three controllers for drying temperature control based on this system were carried out to verify the effectiveness of the INN-PID controller for temperature control. The STM32F407 microcontroller was selected as the control element. The temperature sensor signal is fed into the microcontroller through the I/O port. The microcontroller outputs a variable voltage signal according to the current temperature target value, which adjusts the output frequency of the inverter to change the power of the infrared heating tube and ultimately the temperature of the chamber. The infrared heating tube is rated at 2.1 kW and 380 V, and the temperature sensor is a DS18B20 digital temperature sensor with an accuracy of ±0.1 °C. A temperature adjustment span of 5 °C was chosen to fully verify the performance of the three controllers. The temperature regulation test protocol was set at 50–55 °C combined with the temperatures commonly used in variable-temperature drying processes.

### 2.5. Variable-Temperature Drying Experiment for Cantaloupe Slices

#### 2.5.1. Materials

Fresh, undamaged cantaloupes (variety Xizhou honey No. 17) were purchased locally (Shihezi, Xinjiang) as experimental material. The same batch of cantaloupes with moderate maturity was selected to ensure uniformity in size and weight (mean length, diameter and weight = 32.32 ± 0.27 cm, 17.46 ± 0.21 cm and 4.63 ± 0.29 kg, respectively). The samples were stored in a refrigerator at 4 ± 1 °C. Prior to testing, the cantaloupe was peeled, deseeded and sliced into 30 × 50 × 7 mm slices. The moisture content of fresh cantaloupe was determined by drying the slices in a hot air oven (DHG-9070A, Yiheng Scientific Instrument Co., Ltd., Shanghai, China, with an accuracy of ±0.1 °C) at 105 °C for 24 h [31]. The average initial moisture content of cantaloupe was 90.24% (wet basis).

#### 2.5.2. Experimental Design

Drying experiments on cantaloupe slices were carried out in the air impingement dryer. The drying temperature in the experiment is referred to as the temperature of the hot air emitted from the nozzles. The air velocity is the speed of the hot air emitted from the nozzles, which is 4 m/s. The nozzles have an internal diameter of 10 mm, and the number of nozzles is 18. Figure 1 shows the distribution of the nozzles. The distance from the material to the nozzle is 140 mm.

At present, two kinds of complex variable-temperature drying processes exist, LVT and variable temperature with a controlled drying rate. The complex drying environment in the air impingement dryer makes realising the online measurement of water content difficult. Thus, the linear variable-temperature drying experiment was conducted in this study. LVT drying is a drying process where the drying temperature is linearly related to time. Experiments were carried out on cantaloupe slices at constant temperatures (50 °C, 55 °C, 60 °C and 65 °C) and LVTs. Initial experiments on cantaloupe slices showed that the drying rate was extremely slow when the temperature was below 40 °C. When the drying temperature was above 65 °C, the heat-sensitive substances (vitamin C) in cantaloupe slices were severely lost, so the range of LVT drying was determined to be 40 °C–65 °C. The LVT drying time was set at 250 min based on the initial experiments to ensure that the LVT drying was completed before the material reached the target moisture content. Moreover, after the LVT was completed, the drying was carried out at a constant drying temperature of 65 °C until the end of the experiments.

With improved living standards, people’s requirements for the appearance (colour and shape) and internal (hardness, nutritional composition) qualities of dried products are constantly improving. Colour can be divided into brightness and colour difference. As the primary index of consumers to evaluate dried products, colour has a great impact on the market value of dried cantaloupe slices. As a typical parameter describing the texture properties of dried fruits and vegetables, chewiness reflects the edible taste of dried fruit and vegetable products and has a great impact on the acceptance of consumers [32]. Lower chewiness indicates that dried products are more easily chewed during consumption. As a thermal process, drying can be destructive to nutrient substances with heat sensitivity in the material. As one of the heat-sensitive substances, vitamin C has low stability and is easily decomposed during drying [33]. In addition, changes in the vitamin C content can indirectly reflect changes in the content of other heat-sensitive substances. Drying is one of the most energy-intensive industrial operations. Efficient drying plays an important role in achieving low carbon and sustainable development. Process optimisation can lead to substantial energy savings, even small improvements in the energy efficiency of drying [1]. In summary, the drying experimental results were evaluated comprehensively with the brightness (*L* value), colour difference (Δ*E*), vitamin C content, chewiness, drying time and energy consumption (EC) as evaluation indexes.

#### 2.5.3. Drying Time and EC

Drying time is the time taken to dry cantaloupes to a target moisture content of 12% (wet basis). The approximate time to reach the target moisture content is predicted based on the drying characteristics of cantaloupe slices. The cantaloupe slices were weighed using an electronic balance (BSM-5200.2, Shanghai Joujing Electronic Technology Co., Ltd., Shanghai, China), and the wet basis moisture content of the cantaloupe slices, *M_t_*, was calculated following Equation (27) [34]. If the target moisture content of the cantaloupe slices was reached, then the drying was stopped; if not, then the drying continued.

(27) $$M_{t}=\frac{m_{t}-m}{m_{t}}\times 100\%$$

where *m_t_* is the mass of a cantaloupe slice at time *t* and *m* is the dry matter mass of cantaloupe slices.

The electricity consumption of the dryer is measured in kilowatt-hour metres, and the EC is calculated using Equation (28).

(28) EC=3.6×W

where *EC* is the energy consumption (MJ) and *W* is the electricity consumption (KW·h).

#### 2.5.4. Colour

The surface colour of cantaloupe slices was measured using a colour meter (Model CR-410, Konica Minolta, Tokyo, Japan). The standard illuminant is set to D65, and the viewing angle is set to 2°. Before the measurement, the colour meter must be calibrated with a standard black–white plate. The colour parameter L^*^ indicates the brightness of the material, with a^*^ indicating red and green, whereas b^*^ indicates yellow and blue. The total colour difference (∆*E*) between dehydrated and fresh cantaloupe slices was calculated in Equation (29) [35].

(29) $$\Delta E=\sqrt{{\left(L^{*}-L_{0}^{*}\right)}^{2}+{\left(a^{*}-a_{0}^{*}\right)}^{2}+{\left(b^{*}-b_{0}^{*}\right)}^{2}}$$

where L^*^, a^*^ and b^*^ are colour parameters of dehydrated cantaloupe slices. The mean values of $L_{0}^{*}$, $a_{0}^{*}$ and $b_{0}^{*}$ for fresh cantaloupe slices were 72.02, 9.06 and 31.07, respectively.

#### 2.5.5. Chewiness

Chewiness is the product of hardness, cohesiveness and springiness. The chewiness of cantaloupe slices was measured using a texture analyser (Model TA-XT plus, Stable Micro System, Godalming, UK). The test parameters were set as follows: using the P/50 probe, the probe speed was 1, 0.5 and 1 mm/s before, during and after the test, respectively. The deformation of the cantaloupe slices under pressure was 50%. The two compression pauses were 5 s. The results of 10 measurements for each group of samples were averaged. Niu Yubao [32] described the specific method for the chewiness test.

#### 2.5.6. Vitamin C Content

The vitamin C content of dehydrated cantaloupe slices was measured through titration with 2,6-dichloroindophenol (0.01 g/100 g solution) [36]. The crushed dehydrated cantaloupe sample (10 g) was diluted to 100 mL with an oxalic acid solution (20 g/L) in a 100 mL volumetric flask, extracted using an ultrasonic wave (KQ52000DE, Kunshan Ultrasonic Instruments Co., Shanghai, China) for 15 min and filtered. The filtrate (10 mL) was titrated using 2,6-dichloroindophenol (0.01 g/100 g solution) until the filtrate turned pink and did not fade within 15 s. The titration solution consumed was recorded and repeated three times. In addition, a 20 g/L solution of oxalic acid was titrated in the same way. The results of vitamin C content were expressed as mg/100 g of dehydrated cantaloupe.

#### 2.5.7. Microstructure

The dried cantaloupe slices under different drying temperature modes were processed to the appropriate size with a blade and fixed on the sample table with double-sided tape. The sample was placed under an electron microscope (Model SU-8010, Hitachi, Japan) and then observed.

## 3. Results and Discussion

### 3.1. Simulation Results and Analysis

**Figure 7:**
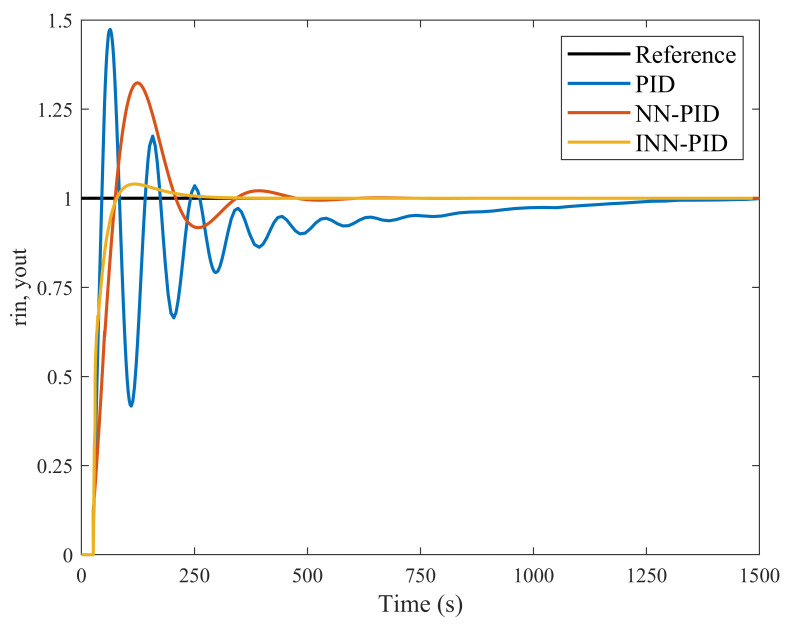
Comparison of the control effect of the three controllers.

As shown in Figure 7, significant differences exist in the dynamic performance of the three controllers. As presented in Table 1, although the peak times of the conventional PID controller are significantly shorter than the other two controllers, the regulation times and maximum overshoot of the conventional PID controller indicate that the conventional PID controller has an extremely slow regulation speed and poor stability during the regulation process. Compared with conventional PID controllers, the NN-PID controller has more improvement in regulating time and maximum overshoot. However, its control effect does not meet the design expectations. The INN-PID controller has improved further compared with the NN-PID controller, as the neural network has been improved in several ways. The regulation time and overshoot were reduced by 73.37% and 85.36%, respectively, compared with the NN-PID controller. Although the INN-PID controller has the largest peak time, Figure 7 shows that almost no oscillation exists in the adjustment process of the INN-PID controller, and after reaching the target value, the controller rapidly stabilises. Taken together, the dynamic performance of the INN-PID controller is significantly better than the other two controllers and meets the system design requirements.

### 3.2. Drying Temperature Control Experiments with Three Controllers

**Figure 8:**
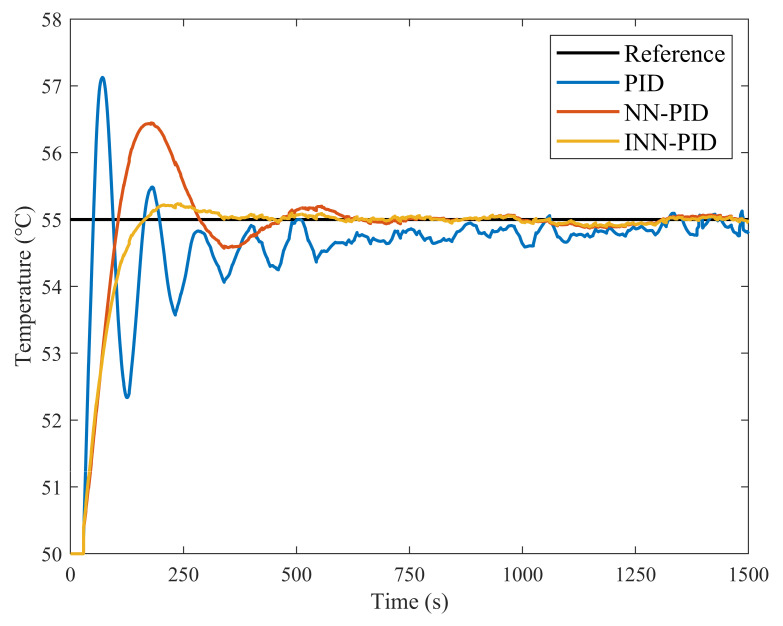
Comparison of drying temperature control effect of three controllers.

As shown in Figure 7 and Figure 8, the actual temperature control results follow approximately the same pattern as the simulation results, confirming the validity of the simulation results and the accuracy of the mathematical model for temperature control. Unlike the simulation results, many disturbances occurred during the experiment, which caused many small fluctuations in the actual temperature profile. Figure 8 and Table 2 again present the superiority of the INN-PID controller under the action of step signals. Engineering practice shows that the step input is a relatively severe operating condition for the control system. The drying temperature control effect of the system under the action of a step signal is good. When the input signal of the system is in other forms, such as the ramp signal, the control effect is also satisfactory. The INN-PID controller can quickly and effectively regulate the temperature of the inner chamber of the air impingement dryer during variable-temperature drying.

### 3.3. Drying Experimental Results and Analysis

**Figure 9:**
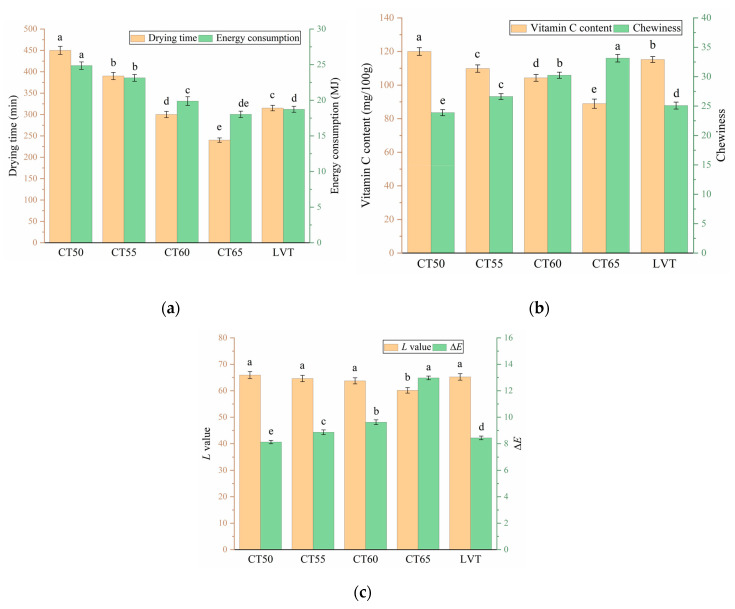
Comparison of six indexes under different temperature modes (**a**–**c**). Note: different letters of columns of same colour indicate significant differences between the mean values (*p* < 0.05).

Figure 9 shows the comparison of the *L* value, Δ*E*, vitamin C content, chewiness, drying time and EC for five temperature modes: constant temperature of 50 °C (CT50), 55 °C (CT55), 60 °C (CT60), 65 °C (CT65) and LVT. The *L* values of CT50, CT55, CT60 and LVT were not significantly different, but the *L* value of CT65 was significantly lower than that of the other four groups (*p* < 0.05). The reason is that the reaction of reducing sugars and amino acids in cantaloupe slices under aerobic and high-temperature conditions produces darker components, reducing the *L* value of the product [37]. In the constant-temperature drying experiment, Δ*E* increased significantly with the increasing temperature. The reason is the increase in enzymatic browning results in heavier colour deterioration [38]. In the LVT temperature model, the material was at high temperature for a shorter period, and therefore, Δ*E* was at a lower level, between CT50 and CT55.

**Figure 10:**
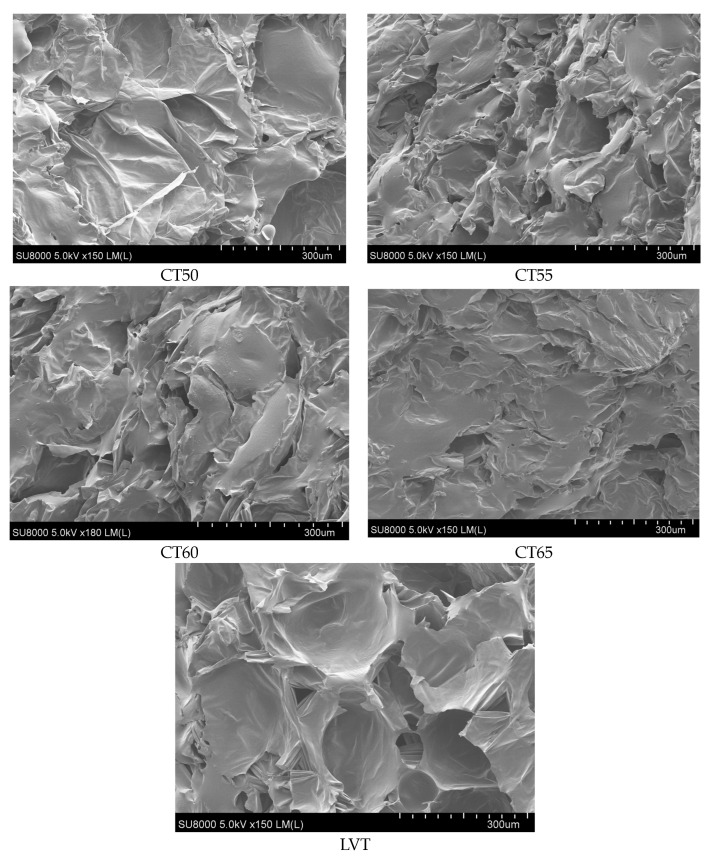
Electron microscopic images of materials with different temperature modes.

In Figure 9b, the vitamin C content decreases with an increasing drying temperature in constant-temperature drying. The reason for this is the thermal sensitivity and instability of vitamin C, which is easily degraded during drying, particularly at high temperatures [39]. Yubao Niu [40] also found similar results when studying the nutritional quality changes of winter jujube slices during the drying process. The materials in the LVT drying mode were exposed to high temperatures for a short time, vitamin C was not degraded too much and the vitamin C content was at a high level. In constant-temperature drying, chewiness increased with the increasing drying temperature, similar to the findings of Özge SÜFER [41] during the convective drying of onion slices. Figure 10 shows that the higher the drying temperature, the more severe the collapse of the tissue structure of the cantaloupe slices, the more compact the surface tissue and the more severe the hardening. The surface hardening makes dried cantaloupe slices harder to chew and increases chewiness. In particular, crusting was observed on the surface of the cantaloupe slices at 65 °C. The reason for this is that at the beginning of the drying process, the moisture content of the material is high, and the rate of water loss on the surface of the material is fast. Moreover, the internal moisture of the material is not able to diffuse to the surface of the material quickly, which will lead to hardening and crusting on the surface of the material. The increase in the drying temperature aggravates this phenomenon. In the LVT mode, the initial drying temperature is low, and the rate of water loss from the material surface is not high. As the moisture content of the material decreases, the temperature is slowly increased, thereby avoiding this phenomenon.

Figure 9c shows that the drying time decreases with increasing temperature in constant-temperature drying. According to the Stephan–Boltzmann law, this case is caused by an increase in the thermal radiation intensity and the moisture transfer rates [42]. The reduced drying time also means that the equipment runs less. Therefore, EC and the drying time have the same regularity. The drying time and EC of the LVT are at a low level. This case may be because the material is loosely organised and the channels for water diffusion to the outside are completed, which accelerates the rate of water loss and reduces the drying time and EC. The drying time and EC of the LVT temperature mode were significantly lower than those of CT50 and CT55 and were at a lower level.

Therefore, the LVT temperature mode is an effective and feasible drying method with evident advantages. This mode ensures the quality of the material and reduces the drying time and EC, based on the comprehensive evaluation of the five indicators.

## 4. Conclusions

This study fits a mathematical model for drying temperature control in the air impingement dryer. The neural network is improved by optimising the initial weights, adding a variable learning rate algorithm, adding a momentum method and optimising the input parameters. The theoretical derivation of the INN-PID algorithm is completed. A controller based on the INN-PID algorithm was designed. In addition, a drying temperature precision control system in the air impingement dryer was set up. The dynamic performance of the PID, NN-PID and INN-PID controllers was simulated in MATLAB software with the unit step signal as input. The simulation results show that the INN-PID controller has a peak time of 132.78 s, a regulation time of 80.37 s and a maximum overshoot of 3.99%. The INN-PID controller is significantly better than the other two controllers in terms of the control accuracy and regulation time. A drying temperature control experiment was carried out to verify the performance of the three controllers in a precise drying temperature control system. The actual temperature control results showed approximately the same pattern as the simulation results, which confirmed the validity of the simulation results and the accuracy of the mathematical model. The INN-PID controller meets the temperature control requirements of the variable-temperature drying process. An LVT drying experiment and a constant-temperature drying experiment were carried out on cantaloupe slices based on this system. The results were evaluated using the *L* value, Δ*E*, vitamin C content, chewiness, drying time and EC as evaluation indicators. Under the LVT drying mode, the *L* value of dried cantaloupe slices was 65.23, Δ*E* was 8.43, vitamin C content was 115.23 mg/100 g, chewiness was 25.08, drying time was 315 min and EC was 18.73 MJ. The LVT is an effective drying mode as it ensures the quality of the material and reduces the drying time and EC, which is worthy to be applied in the production of dried cantaloupe slices. The precise temperature control system based on the INN-PID controller meets the temperature control requirements of the variable-temperature drying process and provides technical support for further research into the variable-temperature drying process. This study proves that the variable-temperature drying process is more effective than the constant-temperature drying process and deserves further research. The LVT drying model improved in this study provides a reference for producing dried cantaloupe slices in the drying industry.

## Figures and Tables

**Table 1:** Comparison of the dynamic performance of three controllers.

Controller Type	Peak Time (s)	Regulation Time (s)	Maximum Overshoot
PID	63.53	797.48	47.36%
NN-PID	123.86	301.82	32.37%
INN-PID	132.78	80.37	3.99%

**Table 2:** Comparison of the dynamic performance of three controllers in the experiments.

Controller Type	Peak Time (s)	Regulation Time (s)	Maximum Overshoot
PID	70.44	1154.79	42.52%
NN-PID	172.64	401.19	28.90%
INN-PID	237.37	134.91	4.74%

## Data Availability

The data presented in this study are available on request from the corresponding author.
